# Influence of glucocorticoids on the osteogenic differentiation of rat bone marrow-derived mesenchymal stem cells

**DOI:** 10.1186/1471-2474-15-239

**Published:** 2014-07-15

**Authors:** Da-An Zhou, Hong-Xin Zheng, Cheng-Wen Wang, Dan Shi, Jian-Jun Li

**Affiliations:** 1 Department of Spinal and Neural Function Reconstruction, School of Rehabilitation Medicine of Capital Medical University, China Rehabilitation Research Center, No. Jiaomeibei Road Fengtai District, Beijing 100068, China; 2 Department of Rehabilitation, the 3rd Affiliated Hospital of Liaoning Medical University, Jinzhou 121000, China; 3 Liaoning University of Traditional Chinese Medicine, Shenyang 110032, China

**Keywords:** Glucocorticoid, Osteogenic differentiation capacity, Osteoprotegerin, RANKL, Klotho gene

## Abstract

**Background:**

Glucocorticoid has been used extensively in clinical applications, because of its several pharmacologic actions, which include immunosuppression, anti-inflammation, anti-shock, and relief of asthma. However, the long-term or high-dose application of glucocorticoid can induce adverse effects such as osteoporosis, which is known in this case as glucocorticoid-induced osteoporosis (GIOP). It is a secondary osteoporosis that results in easy fracturing, and even disability. Therefore it became a thorny issue.

**Methods:**

The rat model of glucocorticoid-induced osteoporosis (GIOP) was replicated to isolate BMSCs. Rats were assigned into four groups: normal, normal induction, GIOP, and GIOP induction. The growth cycle was monitored by using flow cytometry. Osteogenic differentiation was compared by using alkaline phosphatase (ALP) staining with a modified calcium cobalt method. The quantitative detection of osteoprotegerin and the receptor activator of nuclear factor kappa-B ligand (RANKL) was conducted by using enzyme-linked immunoassay. Finally, renal Klotho mRNA expression was assessed by using RT-PCR.

**Results:**

BMSC proliferation was reduced in GIOP rats. The ALP-positive expression of normal BMSCs to the osteogenic induction solution was stronger than that of BMSCs from GIOP rats (P < 0.01). Osteoprotegerin expression was significantly higher in the normal induction group than in the normal, GIOP (P < 0.01), and GIOP induction groups (P < 0.05). RANKL expression was significantly higher in the normal induction group than in the other groups (P < 0.01) and significantly higher in the normal group than in the GIOP and GIOP induction groups (P < 0.01). RT-PCR analysis showed that renal Klotho mRNA expression was significantly reduced in the GIOP group compared with the normal group (P < 0.01).

**Conclusion:**

BMSC proliferation, osteogenic differentiation, and reactive activity to an osteogenic inductor were reduced in GIOP rats. Klotho mRNA expression decreased during GIOP induction.

## Background

Osteoporosis (OP) is a systemic skeletal disease characterized by low bone mass, increased bone fragility, and high morbidity and mortality. In China, OP is one of three age-related diseases included in the National Key Research Fund. In the United States, the treatment of fractures caused by OP is estimated to cost USD 15 billion [1]. OP can increase the incidence of fractures and is one of the main complications of glucocorticoid (GC) application [2]. This study was carried out in strict accordance with the recommendations in the Guide for the Care and Use of Laboratory Animals of the National Institutes of Health. The animal use protocol has been reviewed and approved by the Institutional Animal Care and Use Committee (IACUC) of Liaoning Medical University.

Osteoblast (OBs) and fat cells originate from bone marrow-derived mesenchymal stem cells (BMSCs) and have the same cell phenotype. When conditions are met, OBs and fat cells increase/decrease inversely [3]. GCs promote and inhibit the differentiation of BMCSs into lipocytes and OBs, respectively, thus leading to coupling imbalance between OBs and osteoclasts. This phenomenon causes bone loss and OP [4]. Therefore, the development of specific drugs to reduce bone turnover rate has become an urgent task. The present study aims to investigate the influence of GC on the osteogenic differentiation of rat BMSCs and determine the pathological mechanism of GCs to establish a foundation for the prevention of glucocorticoid-induced osteoporosis (GIOP) by Chinese medicine.

## Methods

### Animals

A total of 20 Sprague–Dawley rats (half male and half female) aged eight months and weighing 350 ± 10 g were provided by the Laboratory Animal Center of Liaoning Medical University. The rats were assigned equally into four groups: normal (normal BMSCs from rats), normal induction (normal BMSCs from rats + osteogenic induction solution [5,6]), GIOP (BMSCs from GIOP rats), and GIOP induction (BMSCs from GIOP rats + osteogenic induction solution) groups.

### Establishment of the GIOP model

GCs could directly inhibit the osteogenic activity of osteoblasts and reduce the bone formation, leading to loss of bone mass [7]. According to reported methods [8,9], 10 of 20 rats were randomly selected to establish the GIOP models. Dexamethasone was applied into the hind limbs of rats by intramuscular injection at concentrations of 2.5 mg/kg twice a week for 9 consecutive weeks.

### Isolation, cultivation, and subcultivation of BMSCs

A constant volume of the cell culture solution was maintained at 100 mL under sterile conditions. The solution was fully mixed and stored at 4°C. An osteogenic differentiation induction solution composed of 10^-8^ mol/L Dex, 50 μmol/L Vitamin C, 10 mmol/L β-GP, and 10% fetal bovine serum was used to isolate and culture the BMSCs following the whole bone marrow adherence method. Rats were dipped in 75% ethanol for 20 min and anesthetized with chloral hydrate. Under aseptic conditions, the bilateral lower limb femurs of rats were obtained followed by the removal of attached fatty tissues, connective tissues, and periosteum. The femurs were placed in a small aseptic beaker and then moved on a super clean bench. Thereafter, the femurs were stored on a sterile culture dish. After washing with PBS, the bilateral mummification ends in the femur were resected. A total of 5 ml G-DMEM containing 10% FBS was mixed with 0.5 ml of heparin. The marrow cavities of the femurs were rinsed by the mixed solution for three to four times. The flushing fluid was fully beaten followed by cell resuspension with G-DMEM containing 10% FBS. The cells (density was adjusted to 1 × 10^6^ cells/ml) were inoculated in a 25 cm^2^ culture bottle and were then cultured (37°C, 5% CO_2_, saturated humidity). The medium was changed every three days. After the adherent cells reached 80% to 90% fusion, the culture medium was abandoned followed by washing with PBS for three times. The preheated (37°C) digestion liquid containing 0.25% trypsin and 0.02% EDTA was used for digestion at room temperature followed by passage with a ratio of 1:2.

### Determination of BMSC growth cycle and osteogenic differentiation capacity

The passage 3 growth cycle of BMSCs from normal and GIOP rats was monitored by using flow cytometry. Osteogenic differentiation was evaluated by using alkaline phosphatase (ALP) staining with a modified calcium cobalt method. Passage 3 BMSCs from normal and GIOP rats were seeded in a 24-well culture plate at a density of 5 × 10^4^ cells/mL with 6 parallel wells in each group. The induction groups were incubated with the osteogenic differentiation induction solution with a final volume of 2.5 mL in each well. The solution was replaced every 3 days for 21 consecutive days. Glass slides were harvested for ALP staining at 7, 14, and 21 days. To quantify the number of ALP-positive cells and the total number of cells and calculate the positive staining rate, 10 non-overlapping fields of view were randomly selected under a light microscope.

### Determination of OPG and RANKL expression

On day 14, cell supernatants were harvested from each induction group and stored at -20°C. OPG and receptor activator of nuclear factor kappa-B ligand (RANKL) expressions were determined by using ELISA (ELISA USAR&D Systems, USA).

### Determination of Renal Klotho mRNA expression

RT-PCR primers and RNA PCR Kit (AMV) Ver.3.0 kit were sourced from Takara, Dalian (China). The 600 bp DNA ladder marker was obtained from Tianze Genetic Engineering Co. Ltd. (China). Primers were designed by using Primer Premier 5.0 according to the rat Klotho (KL) and β-actin gene sequences determined in GenBank, The PCR products of KL and β-actin gene were 383 and 277 bp in length,respectively. The total RNA was extracted according to the Trizol Reagent instructions (Invitrogen, USA). The first cDNA strand was synthesized according to the Takara RT-PCR kit instructions. The reaction conditions were as follows: 42°C for 30 min → 99°C for 5 min → 5°C for 5 min → store at 4°C. The PCR-amplified products were detected by electrophoresis at 9 V/cm (90 V) for 1 hour with 3 μL of 100 bp standard molecular weight DNA ladder marker as a reference.

### Statistical analysis

Experimental data were expressed as mean ± SD and analyzed by using SPSS 11.0 software. The electrophoresis results were analyzed by using Fluor Chem V 2.0 gel imaging analysis software (Gene Genus, Syngene Inc., USA).

## Results

### Comparison between the BMSC growth cycles for normal and GIOP rats

**Figure 1:**
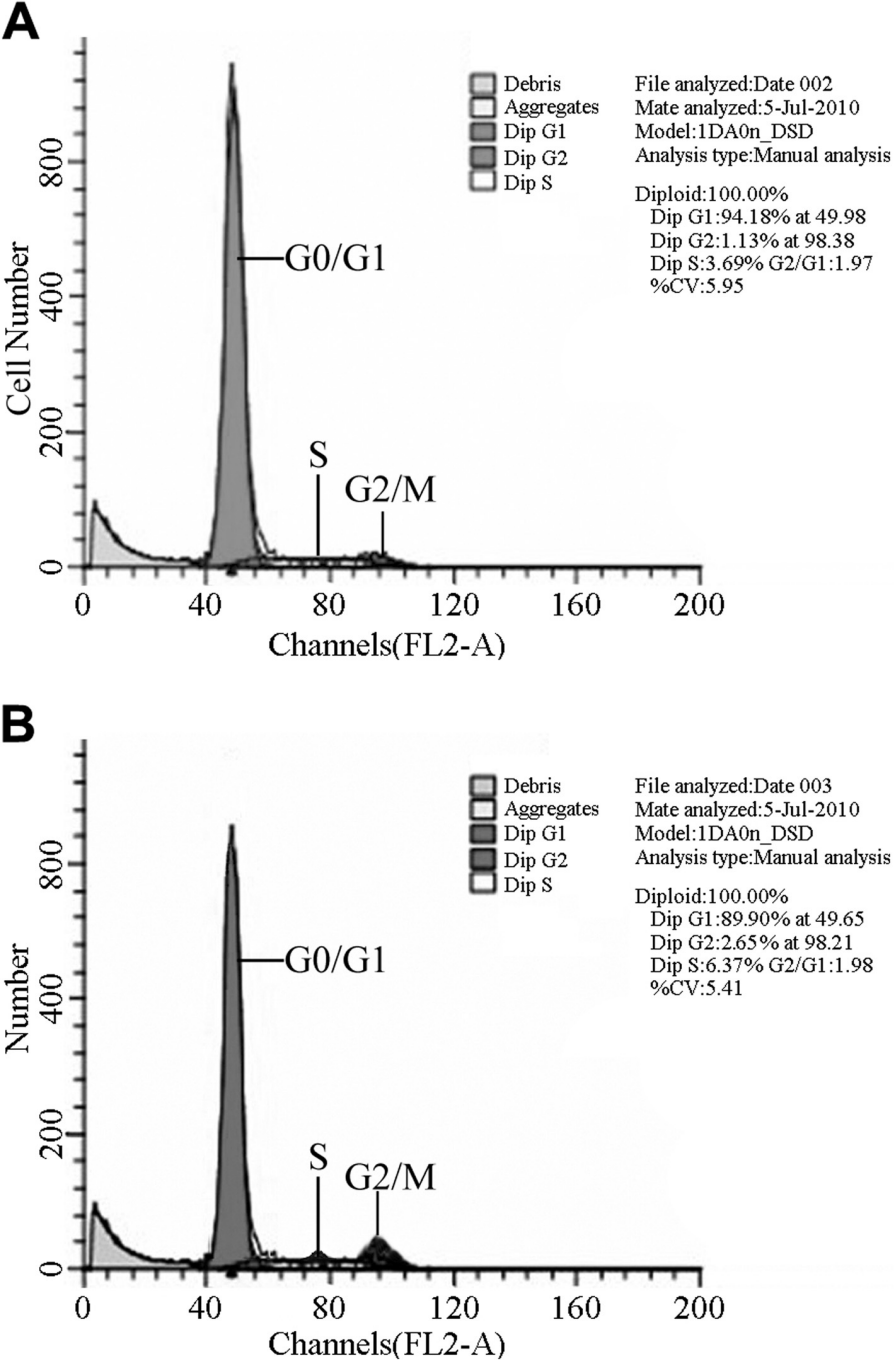
**BMSC growth cycles for normal and GIOP rats. (A)** Normal group; **(B)** GIOP group.

Approximately 10.10% and 5.82% of BMSCs in normal and GIOP rats were arrested at S + G_2_ + M, respectively (Figure 1A and B).

### Comparison between osteogenic differentiation capacities

**Figure 2:**
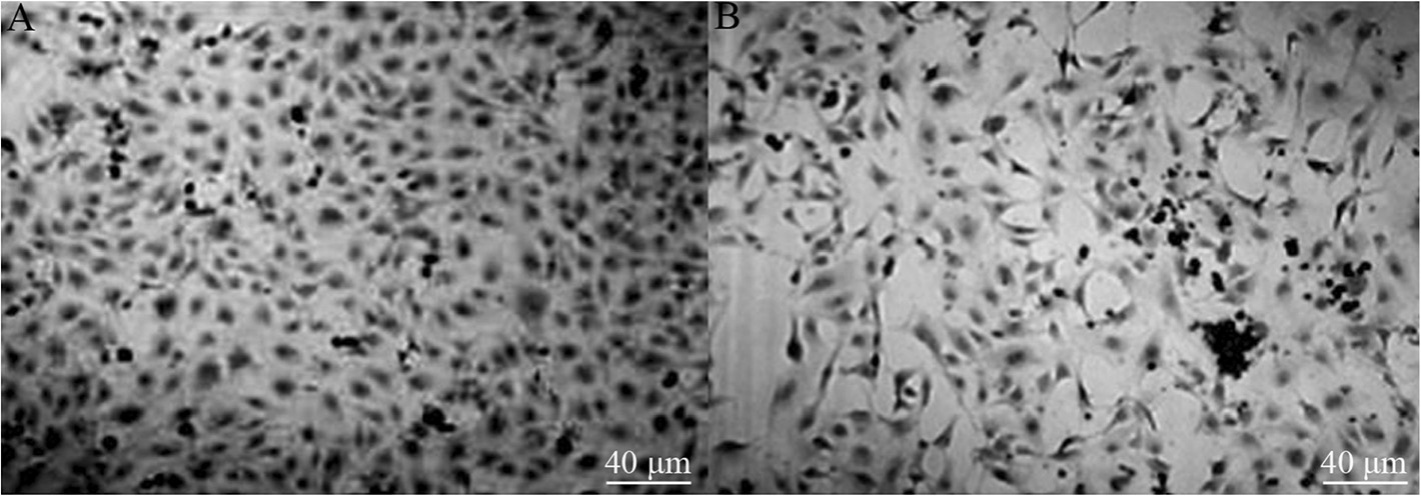
**Osteoblasts after culturing for 14 days (×100). (A)** Normal induction group; **(B)** GIOP group.

**Figure 3:**
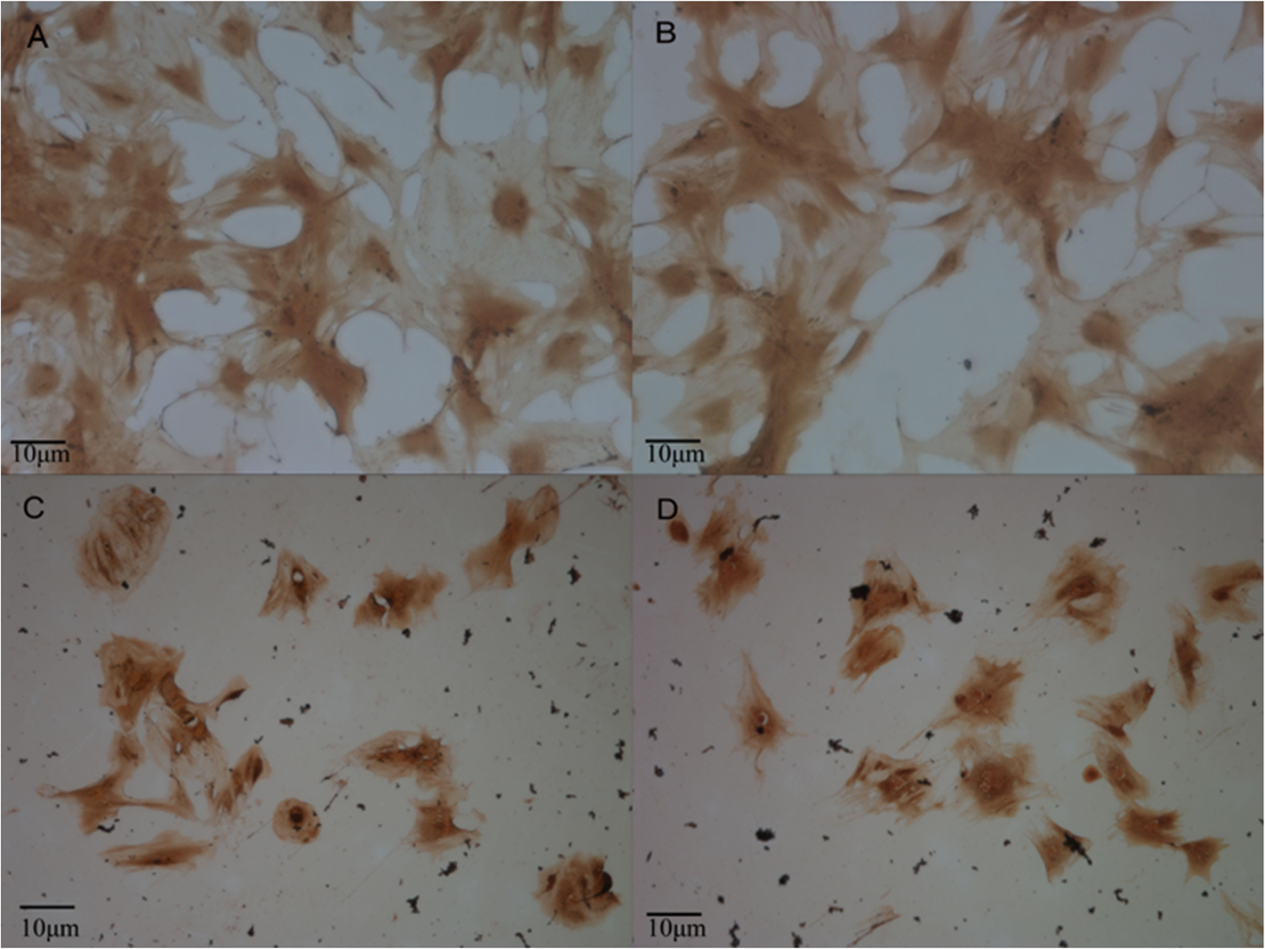
**Comparison between the ALP-positive rates in different groups for 21 days (×400). (A)** Normal group; **(B)** Normal induction group; **(C)** GIOP group; **(D)** GIOP induction group.

The ALP-positive rates at the 7th, 14th, and 21st days in the normal induction group were significantly higher than the normal and GIOP groups, respectively (*P* < 0.01) (Table 1 and Figures 2 and 3). The ALP-positive rates at the 7th and 14th days in the normal induction group were also significantly higher than the GIOP induction group (*P* < 0.01), except for the 21st day of incubation (*P* > 0.05). The ALP-positive rates at the 7th, 14th, and 21st days in the GIOP induction group were significantly higher than in the normal and GIOP groups (*P* < 0.01). The ALP-positive rates at the 14th and 21st days in the normal group were significantly higher than in the GIOP group (*P* < 0.01). These results indicated that the ALP-positive expression in normal and GIOP rats increased with the action of the osteogenic inducer; no obvious differences were observed in the responses of rats in these two groups to the osteogenic inducer at the 21st day.

**Table 1:** **Comparison between the ALP-positive rates in different groups (**$\bar{x}$**± s, n = 3)**

**Group**	**7 d**	**14 d**	**21 d**
Normal	2.3332 ± 0.5774^▲▲△△^	7.0011 ± 0.9882^▲▲△△^	8.3289 ± 1.1632^▲▲△△^
Normal induction	20.3333 ± 1.5275	30.3245 ± 1.5268	30.3245 ± 1.5268
GIOP	1.6668 ± 0.5742^▲▲△△^	2.3298 ± 0.5699^◆▲▲△△^	3.0324 ± 1.0349^◆▲▲△△^
GIOP induction	12.6659 ± 0.5543^▲▲^	21.2969 ± 2.1375^▲▲^	31.6596 ± 1.5468^▲▲^

^◆^*P* < 0.05, ^◆◆^*P* < 0.01, vs. normal group; ^▲^*P* < 0.05,^▲▲^*P* < 0.01, vs. normal induction group; ^△^*P* < 0.05, ^△△^*P* < 0.01, vs. GIOP induction group.

### Comparison between OPG and RANKL expression

OPG expression was significantly higher in the normal induction group than in the normal, GIOP (*P* < 0.01), and GIOP induction groups (*P* < 0.05). OPG expression was significantly higher in the GIOP induction group than in the GIOP (*P* < 0.01) and normal groups (*P* < 0.05). No significant difference was found between the normal and GIOP groups (*P* > 0.05).

RANKL expression was significantly higher in the normal induction group than in the other groups (*P* < 0.01). Furthermore, RANKL expression was significantly higher in the normal group than in the GIOP and GIOP induction groups (*P* < 0.01). RANKL expression was higher in the GIOP induction group than in the GIOP group but no significant differences were observed (*P* > 0.05; Table 2).

**Table 2:** **Comparison between OPG and RANKL expression in different groups (**$\bar{x}$**± s, n = 5)**

**Group**	**OPG (ng/ml)**	**RANKL (pmol/l)**
Normal	16.3092 ± 0.8425^▲▲△^	3.0708 ± 1.2391^▲▲^
Normal induction	23.8044 ± 1.7818	5.0082 ± 1.0355
GIOP	14.1432 ± 2.6551^▲▲△△^	1.6027 ± 0.1123^◆◆▲▲^
GIOP induction	20.1376 ± 3.0025^▲^	2.0739 ± 0.2375^◆◆▲▲^

^◆^*P* < 0.05, ^◆◆^*P* < 0.01, vs. normal group; ^▲^*P* < 0.05, ^▲▲^*P* < 0.01, vs. normal induction group; ^△^*P* < 0.05, ^△△^*P* < 0.01, vs. GIOP induction group.

### Comparison between renal Klotho mRNA expressions in different groups

**Figure 4:**
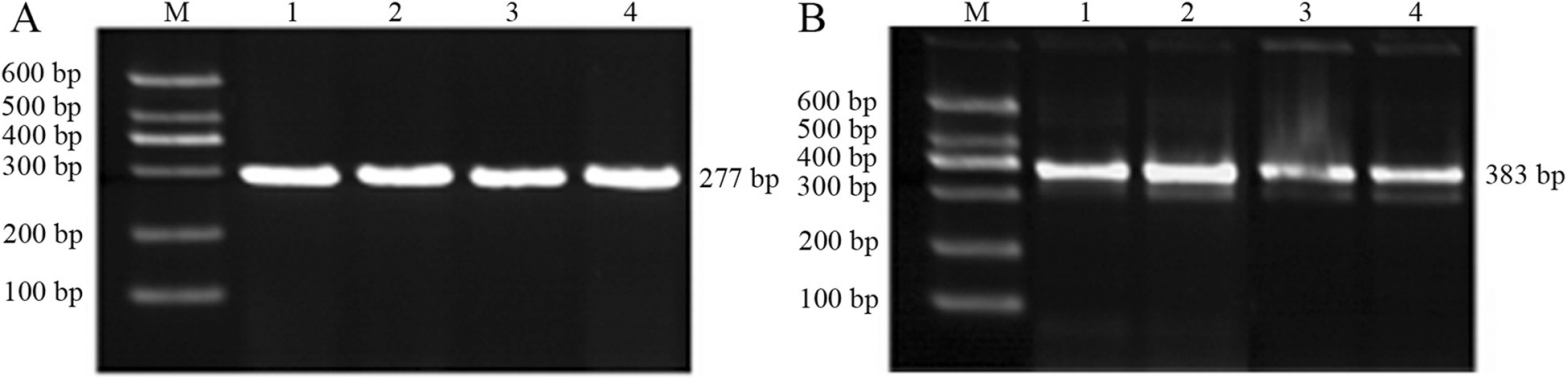
**Electrophoresis of renal Klotho mRNA expression detected by RT-PCR. A)** β-actin gene (277 bp); **B)** KL gene (383 bp). M: DNA Marker DL 600 bp; lanes 1 and 2: normal group; lanes 3 and 4: GIOP group.

Renal tissues from each group were amplified by using target gene and internal reference primers. Two bands with 383 and 277 bp were found as expected (Figure 4). Results from the image analysis software showed that renal KL mRNA expression was significantly reduced in the GIOP group compared with the normal group (*P* < 0.01). Table 3 shows that KL mRNA expression was significantly decreased during GIOP.

**Table 3:** **Influence of glucocorticoid on Klotho mRNA expression (**$\bar{x}$**± s)**

	**Normal group**		**GIOP group**	
Klotho	736	874	538	625
β-actin	1047	1032	1028	1040
Klotho/β-actin	0.702961	0.846899	0.523346	0.600962
$\bar{x}$	0.7765 ± 0.1040		0.5622 ± 0.5657^◆◆^	

^◆^*P* < 0.05, ^◆◆^*P* < 0.01, vs. normal group.

## Discussion

After the transplantation of BMSCs in the GIOP rats, the growth rate of a large proportion of adipocytes and primary cells at three, four, five, and eight days was slower than the growth rate of primary cells from normal rats. Moreover, BMSCs from GIOP rats were worse than the BMSCs from normal rats with poor refraction. Approximately 10.10% of BMSCs in the normal rats were arrested at S + G_2_ + M but only 5.82% of the BMSCs from the GIOP rats were arrested at S + G_2_ + M. Thus, the proliferation of BMSCs from GIOP rats was worse than the proliferation of normal cells.

Serum osteogenic differentiation ALP is an endocellular enzyme that can be used to evaluate the degrees of osteogenic differentiation of various cells and OB functions. Moreover, ALP serves as the relative specific index of the activity of OBs and the capacity of bone tissue calcification [10].

RANKL and OPG are recently discovered members of a superfamily of tumor necrosis factor receptors and ligands. They form the RANKL–RANK–OPG axis, which is the final pathway in the regulation of bone absorption and *in vitro* calcium ion balance, and plays a key role in bone formation and bone resorption coupling [11,12]. A variety of bone metabolism factors regulate bone formation through this axis. Studies demonstrated that OPGs isolated from humans and rats were 94% homologous and that OPG was mainly produced by OBs in bone tissues [3]. Further studies showed that OPG could inhibit osteoclast formation, differentiation, and survival and could induce OB apoptosis [4]. Patients with bone metabolic diseases were observed with RANK and OPG mutations [13]. GCs at physiological concentrations enhance osteoclast function by increasing the expression of parathyroid hormone receptors, whereas excessive physiological doses of GCs promote an increase in the RANKL/OPG ratio, thus accelerating osteoclast generation and aggravating osteoclasia [14].

In this study, we measured ALP, OPG, and RANKL expressions in BMSCs from normal and GIOP rats in the presence or absence of osteogenic induction to compare the osteogenic differentiation capacity in the different groups.

The ALP-positive expression rate results showed that the ALP-positive rates at 7, 14, and 21 days were significantly higher in the normal induction group than in the other groups (*P* < 0.01). The ALP-positive expression rates at 7, 14, and 21 days were significantly higher in the GIOP induction group than in the normal and GIOP groups (*P* < 0.01). Moreover, at 14 and 21 days, the ALP-positive rate was significantly higher in the normal group than in the GIOP group (*P* < 0.05). These results indicated that in the presence of osteogenic inductor, ALP-positive expression increased in BMSCs from both normal and GIOP rats. BMSCs from normal rats showed stronger reactive activity toward the inductor than BMSCs from GIOP rats.

OPG expression in the normal induction group was significantly higher than in the normal, GIOP (*P* < 0.01), and GIOP induction groups (*P* < 0.05). Moreover, OPG expression in the GIOP induction group was significantly higher than in the GIOP (*P* < 0.01) and normal groups (*P* < 0.05). RANKL expression was significantly higher in the normal induction group compared with the other groups (*P* < 0.01). RANKL expression was significantly higher in the normal group compared than in the GIOP and GIOP induction groups (*P* < 0.01). These findings indicated that the osteogenic inductor could stimulate OPG and RANKL expression.

KL is associated with aging and was discovered by Kuro-o *et al.*[15]. The Kl gene in humans and mice are located in chromosome 13 (13 q12) and exhibits high homology (83%) [16]. KL is mainly distributed in the kidneys and brain choroid. KL gene mutation or deletion can result in various phenotypes similar to human aging, such as shortened life span, arteriosclerosis, reduced immune function, and osteoporosis [17]. However, excessive KL expression or exogenous KL supplementation for KL-knockout mice can delay or improve aging symptoms [18]. In the experiments of the current study, renal KL mRNA expression was significantly reduced in the GIOP group compared with the normal group (*P* < 0.01), thus indicating that KL mRNA expression significantly decreased during GIOP. The GC-induced reduction of KL mRNA expression may be involved in the molecular mechanism of bone metabolism.

The results of this study were consistent with previously reported results. In future studies, we will prepare drug-containing sera intervened by kidney reinforcing and marrow-benefiting Chinese medicine to investigate the effects of different blood collecting times and drug-containing sera at different concentrations on the proliferative activity of rat BMSCs based on ALP. We will also determine the optimal BMSC proliferation-promoting dose and bone-directed differentiation concentration of drug-containing sera. We will use the optimal concentration to explore the possible mechanism of kidney-reinforcing Chinese medicine during the osteogenic differentiation of BMSCs.

## Conclusion

BMSC proliferation, osteogenic differentiation, and reaction capacity were decreased by the osteogenic inducer. The expression of the KL gene was significantly reduced during the process of GC-induced GIOP germination.

## Competing interests

The authors declare that they have no competing interests.

## Authors’ contributions

D-AZ participated in the experimental design, carried out isolation of MSC, culture and subculture of SD rats, participated in BMSC osteogenic differentiation testing of normal and GIOP rats, took part in statistical analysis and drafted the manuscript. H-XZ participated in experimental design. DS and C-w Wang participated in the BMSC osteogenic differentiation testing of normal and GIOP rats. J-JL participated in the design of the study, statistical analysis and helped to draft the manuscript. All authors read and approved the final manuscript.

## Pre-publication history

The pre-publication history for this paper can be accessed here:

http://www.biomedcentral.com/1471-2474/15/239/prepub
